# Decomposition of plant‐sourced carbon compounds by heterotrophic betaproteobacteria isolated from a tropical Costa Rican bromeliad

**DOI:** 10.1002/mbo3.344

**Published:** 2016-02-25

**Authors:** Jane Klann, Alexandra McHenry, Carin Montelongo, Shana K. Goffredi

**Affiliations:** ^1^ Biology DeptartmentOccidental CollegeLos AngelesCalifornia

**Keywords:** Betaproteobacteria, bromeliad, Costa Rica, decomposition, rainforest

## Abstract

Betaproteobacteria were the most common isolates from the water‐filled tank of a Costa Rican bromeliad. Isolates included eight species from the orders Neisseriales and Burkholderiales, with close relatives recovered previously from tropical soils, wetlands, freshwater, or in association with plants. Compared to close relatives, the isolates displayed high temperature and comparatively low pH optima, reflecting the tropical, acidic nature of the bromeliad tank. Bromeliad‐associated bacteria most closely related to *Chromobacterium, Herbaspirillum*, and *Aquitalea* were all isolated exclusively at pH 6, while *Ralstonia, Cupriavidus,* and three species of *Burkholderia* were isolated mostly at pH 4. Activity profiles for the isolates suggest pervasive capabilities for the breakdown of plant‐sourced organics, including d‐galacturonic acid, mannitol, d‐xylose, and l‐phenylalanine, also reflecting a niche dominated by decomposition of leaves from the overlying canopy, which become entrained in the tanks. Metabolic activity profiles were overlapping between the Burkholderiales, isolated at pH 4, and the Neisseriales, isolated at pH 6, suggesting that plant material decomposition, which is presumably the underlying process sustaining the tank community and possibly the plant itself, occurs in the tanks at both pH extremes. These results suggest that bromeliad‐associated betaproteobacteria may play an important role in the cycling of carbon in this unusual aquatic habitat.

## Introduction

Plants from the family Bromeliaceae are prominent members of neotropical rain and cloud forests. Bromeliad average density has been estimated at 1000–100,000 ha^−1^ ground area, depending on the study (Sugden and Robins 1979; Richardson 1999). Many species collect large amounts of water in the canopy by the formation of unique foliar arrangements, or “tanks” (Richardson 1999; Benzing 2000), thus these densities represent as much as 50,000 L suspended water in the canopy ha^−1^ (Fish 1983). This creates an unusual aquatic habitat suspended in the canopy, allowing for long‐term retention and decomposition of organic compounds, compared to soil (Pittl et al. 2010; Goffredi et al. 2011a,b). The physiological capabilities of bromeliads, including increased capacity for amino acid and mineral uptake from the tank, are known (Benzing 1970; Winkler and Zotz 2009), but the specific metabolisms of resident bacteria, and their role in carbon cycling in the rainforest, have not been specifically elucidated.

Several studies have investigated the diversity and certain processes driven by microbes within bromeliad tanks. Bacterial abundance has been shown to be 2–5× higher in bromeliad tank debris, compared to ground soils (Pittl et al. 2010) and tank habitat heterogeneity supports a myriad of bacterial residents, including at least 21 bacterial orders/subdivisions, many of which are closely related to bacteria previously found in soil, peat bogs, and stagnant water (Dedysh et al. 2006; Inselsbacher et al. 2007; Rui et al. 2009; Yarwood et al. 2009; Goffredi et al. 2011a). Bromeliad tanks are highly stratified and within a single tank can range from 4 to 6 pH and from 0.5 to 8.0 ppm O_2_, providing many possible niches available within these unusual microbial ecosystems (Laessle 1961; Benzing et al. 1972; Guimaraes‐Souza et al. 2006; Goffredi et al. 2011a,b). Tank pH, in particular, is a major influence on the resident microbial population. In tanks of pH <5, the bacterial community was dominated by Acidobacteria and Alpha‐proteobacteria, while tanks of pH >5 were dominated by Firmicutes and Betaproteobacteria (Goffredi et al. 2011a).

Given the unique nature of the bromeliad tank habitat and the ecological importance of bromeliads in general, it is compelling to further examine specific associated bacterial community members and the possible influences they may have on nutrient cycling. To that end, this study sought to examine the specific capabilities of bromeliad‐associated bacteria, with emphasis on heterotrophic degradation of organic matter, and environmental tolerances. Eight bacterial representatives from three major families within the betaproteobacteria were isolated and characterized, taxonomically and physiologically, from a tank water sample collected from a Costa Rican bromeliad (*Werauhia gladioliflora*). It is expected that these, and other, bromeliad‐associated bacteria play important roles in cycling recalcitrant carbon in the rainforest.

## Methods

### Isolation

**Figure 1:**
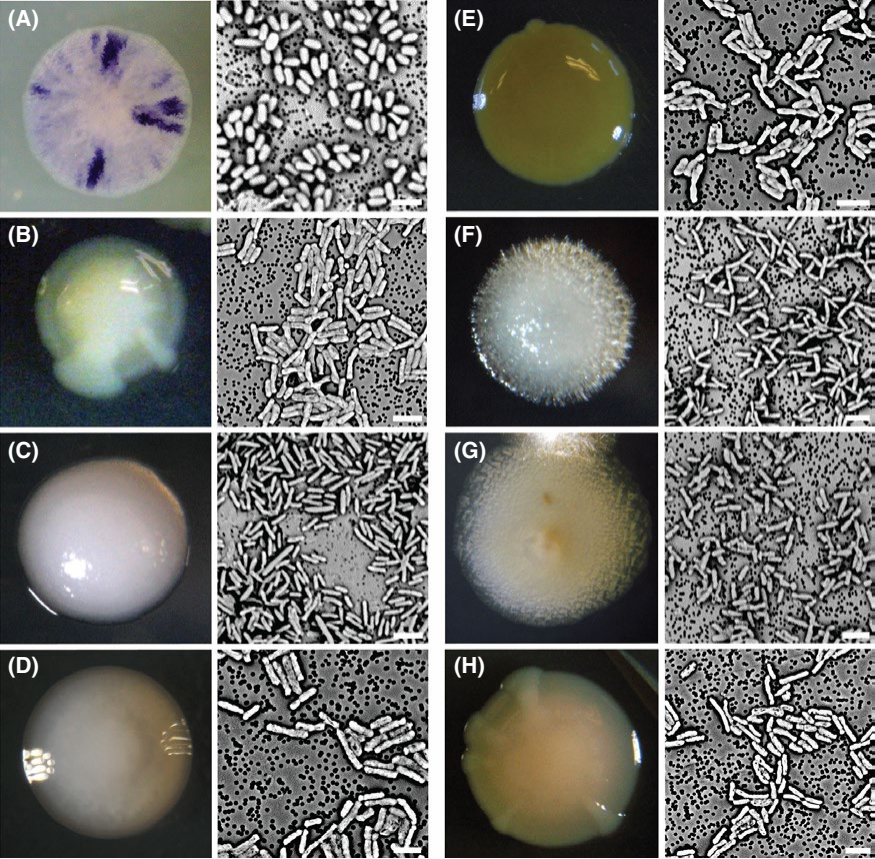
Colony and cell morphology of bromeliad‐associated bacterial isolates. (A) Isolate *Br4*, related to *Chromobacterium*. (B) Isolate *Br23*, related to *Aquitalea*. (C) Isolate *Br2*, related to *Cupriavidus*. (D) Isolate *Br27*, related to *Ralstonia*. (E) Isolate *Br3*, related to *Burkholderia*. (F) Isolate *Br19*, related to *Burkholderia*. (G) Isolate *Br6*, related to *Burkholderia*. (H) Isolate *Br24*, related to *Herbaspirillum*. Image at left of each letter is the colony morphology at 7 days of growth. Image at right of each letter is the individual cell morphology taken via scanning electron microscopy. The specific scales were not originally noted for colony sizes. Bars for the SEM images, 2 *μ*m.

Tank water, with an initial pH of 5.0, was obtained from a bromeliad (*Werauhia gladioliflora*) at La Selva Biological Research Station, Costa Rica, in January 2012. Bacteria were cultivated by streaking serial dilutions on Standard Methods Agar (SMA) of pH 4 or 6. Plates were incubated at 30°C for 1–3 days under aerobic conditions. Single colonies were selected based on unique morphological characteristics (Fig. 1) and further purified by standard T‐streak. The number of viable bacteria in the starting sample was estimated by plating serial dilutions (10^−1^, 10^−2^, and 10^−3^) on Nutrient Agar. Colony‐forming units were counted following incubation for 3 day at 30°C.

### Morphology

Individual cell morphology was determined via scanning electron microscopy (SEM; Fig. 1). Bacterial samples for SEM were initially fixed in 3% glutaraldehyde in 0.1mol L^‐1^ cacodylate for 72 h at 4°C. Samples were then pulled onto a 0.22 *μ*m polycarbonate filter (Millipore, Billerica, MA), washed in a graded ethanol series (50%, 75%, and 100%) and placed in hexamethyldisilazane for 1 h at room temperature. Filters were then mounted, palladium‐coated (Hummer VI, Union City, CA), and visualized using a Phenom desktop SEM (FEI Instruments, Hillsboro, OR).

### DNA extraction, PCR amplification, and 16S rRNA sequencing

**Figure 2:**
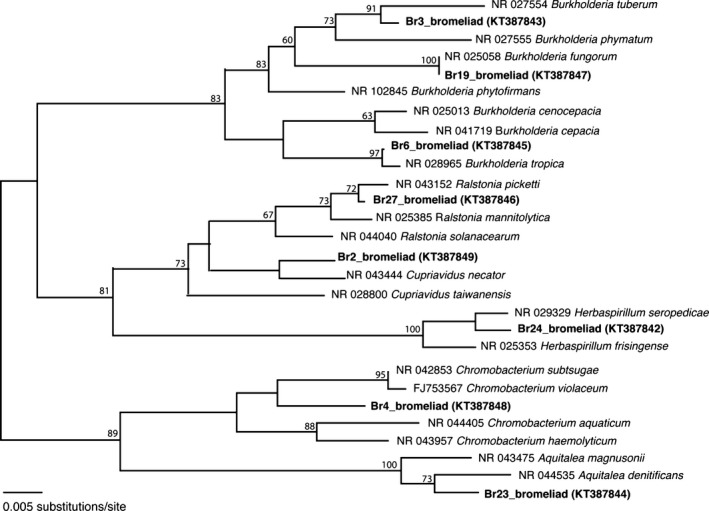
Phylogenetic relationships among betaproteobacteria associated with a Costa Rican bromeliad, relative to selected cultured representatives in public databases, based on sequence divergence within the 16S rRNA gene. (A) neighbor‐joining tree with Kimura two‐parameter distances is shown. The numbers at the nodes represent maximum parsimony bootstrap values from 1000 replicate samplings (only values >60% are shown).

Genomic DNA from each isolate was extracted using the Qiagen DNeasy kit (Qiagen, CA) according to the manufacturer's instruction. 16S rRNA genes were amplified using primers 27F and 1492R, according to Lane 1991; Successful 16S rRNA gene products were cleaned with MultiScreen HTS plates (Millipore Corporation) and sequenced at Laragen, Inc. (Culver City, CA). Closest relatives were identified using BLASTn (Altschul et al. 1997). Sequences were assembled, edited, and aligned using Sequencher v4.10.1 (GeneCodes Corp, Ann Arbor, MI). For bromeliad isolates and closest relatives, neighbor‐joining analysis was used to show phylogenetic relationships (Fig. 2), while maximum parsimony analysis was performed using the heuristic search option with 1000 bootstrap replicates to assign confidence levels to nodes (using PAUP*4.0b10; Swofford 1998). GenBank accession numbers for 16S rRNA sequences obtained in this study are KT387842–KT387849.

### Metabolic characteristics

Amylase function was determined by streak of bacterial isolates on agar plates enriched with starch. After 2 days at 30°C, iodine was added and a zone of clearing was observed. Protein hydrolysis was assessed, via a zone of clearing, on a skim milk plate containing casein. Additional carbon assimilation capabilities were tested using EcoPlates (Biolog Inc., Hayward, CA). Arrays were set up according to manufacturer's instruction using a bacterial suspension with 90% transmissivity, as determined by a Spectronic 20 spectrometer (Milton Roy Company, Warminster, PA). Biolog EcoPlates were incubated at 30°C in the dark and measured intermittently for 12–60 h for changes in absorbance at 590 nm, using an EL800 Microplate Reader (Bio‐Tek Instruments, Winooski, VT). Two statistical comparisons of phenotype patterns among isolates and strains were performed. A hierarchical cluster analysis of bacterial phenotypes, based on Bray–Curtis similarity resemblance (presence/absence, single linkage) of the sum of 19 carbon substrate utilization capabilities, was conducted using Primer v6 (Fig. 4; Clarke and Gorley 2006). Additionally, a *Z*‐test for two population proportions was conducted to determine significance between specific growth and metabolic capabilities of the bromeliad‐associated isolates (assigned as population 1) and closely related strains (assigned as population 2; Table 1).

**Table 1:** Select growth conditions and phenotypic characteristics of bromeliad tank isolates in comparison to other closely related strains

Isolate/Strain	37°C	42°C	pH 4	pH 5	Mannitol	l‐phenyl	Xylose
*Br3*	+	+	+	+	+	+	—
*Br19*	**+**	**+**	**+**	**+**	**+**	**+**	**+**
*Br6*	+	+	+	+	+	+	+
*Br2*	+	+	**+**	**+**	+	**+**	**+**
*Br27*	+	+	+	+	+	+	—
*Br4*	+	+	−	+	+	+	+
*Br23*	+	−	−	+	+	**+**	**+**
*B. tuberum*	+	−	na	na	+	na	na
*B. fungorum*	+	−	na	na	+	na	+
*B. tropica*	+	−	−	+	+	+	+
*B. mimosarum*	+	−	na	na	+	+	+
*C. necator*	+	−	−	−	−	na	−
*C. pauculus*	+	+	na	na	−	na	−
*C. taiwanensis*	+	−	na	na	−	na	−
*R. picketti*	+	+	na	na	−	+	+
*R. mannitolytica*	+	+	na	na	+	+	+
*C. subtsugae*	+	−	−	−	−	−	−
*C. aquaticum*	−	−	−	+	−	+	−
*C. violaceum*	−	−	−	−	−	−	na
*C. piscinae*	−	−	−	+	−	−	−
*A. denitrificans*	+	−	−	+	−	−	na
Isolate proportion	1.00	0.86	0.71	1.00	1.00	1.00	0.71
Strain proportion^a^	0.79	0.21	0.00	0.57	0.36	0.56	0.45
*Z*‐score	1.323	2.806	2.789	1.954	2.806	2.037	1.081
*P* value^b^	0.1868	**0.0049**	**0.0053**	**0.0512**	**0.0049**	**0.0414**	0.2801

Strain references: *Burkholderia tuberum* (Vandamme et al. 2002). *B. tropica* (Reis et al. 2004; Aizawa et al. 2010), *B. mimosarum* (Chen et al. 2006), *B. fungorum* (Coenye et al. 2001), *B. phytofirmans* (Sessitsch et al. 2005). *Cupriavidus necator* (Makkar and Casida 1987); *C. pauculus* (Vandamme et al. 1999; Vaneechoutte et al. 2004); *C. taiwanensis* (Chen et al. 2001; Vaneechoutte et al. 2004); *Ralstonia picketti* (Coenye et al. 2003); *R. mannitolytica* (De Baere et al. 2001; Coenye et al. 2003). *Chromobacterium subtsugae* (Martin et al. 2007); *C. aquaticum* (Young et al. 2008; Kampfer et al. 2009); *C. violaceum* (Martin et al. 2007; Young et al. 2008); *C. piscinae* (Kampfer et al. 2009), *Aquitalea denitrificans* (Lee et al. 2009).

a Proportion calculated for only those strains with known values.

b Based on *Z*‐test for 2 population proportions, with two‐tailed hypothesis.

l‐phenyl, l‐phenylalanine; na, not available from previous studies. bold values denote significance with P values < 0.0512

### Temperature and pH tolerance studies

The temperature ranges for growth were determined visually by 5‐day incubations at 23, 30, 37, 42, and 47°C on SMA plates. The pH ranges were determined via 5‐day incubations at 30°C in Tryptic Soy Broth of incremental pH values between 4 and 7.5, adjusted with 1N HCl or 1M NaOH. Growth was determined via decreases in transmissivity of the suspension, as measured via a Spectronic 20 spectrometer at OD_600_ (Milton Roy Company, Houston, TX).

## Results

### Identity of bromeliad‐associated bacterial isolates

The original Costa Rican bromeliad (*Werauhia gladioliflora*) tank water sample had a total aerobic microbial cell count of 3.2 × 10^5^ c.f.u. mL^−1^ of tank water. Thirty‐five of 41 isolates recovered were members of the betaproteobacteria, five were members of the genus *Bacillus* (Firmicutes) and one was a member of the genus *Roseomonas* (Alpha‐proteobacteria). Betaprotoeobacteria isolates comprised eight distinct types (with representatives shown in Fig. 1). Their phylogenetic relationships are shown in Figure 2; three members of the genus *Burkholderia* (order Burkholderiales) most closely related to *B. tuberum* (*Br3*, 98% similarity in 16S rRNA), *B. fungorum* (*Br19*, 100% similarity)*,* and *B. tropica* (*Br6*, 99% similarity), one closely related to *Cupriavidus necator* (*Br2*, 97% similarity in 16S rRNA; order Burkholderiales), one most closely related to a species within the sister genus, *Ralstonia picketti* (*Br27,* 99% similarity, order Burkholderiales), one most closely related to *Herbaspirillum seropedicae* (*Br24*, 99% similarity in 16S rRNA, order Burkholderiales), one most closely related to *Chromobacterium subtsugae* (*Br4*, 99% similarity in 16S rRNA; order Neisseriales), and one isolate was most closely related to *Aquitalea denitrificans* (*Br23,* 99% similarity, order Neisseriales).

### Phenotypic characterization of bromeliad‐associated bacterial isolates

All isolates were observed to grow at temperatures ranging from 23 to 42°C (Tables S1–S3), with the exception of one isolate *Br2*, related to *Cupriavidus*, which grew at 47°C. Growth at 42°C was a significantly distinct feature of the bromeliad‐associated isolates (*P* = 0.0049; Table 1), compared to related strains. In general, the Burkholderiales were isolated at pH 4 (and grew well at a range of 4–7), while the Neisseriales were isolated at pH 6, and grew only above pH 5 (Tables S1–3). Similarly, the ability to grow at pH 4 and 5 was more common for the bromeliad‐associated isolates (*P* = 0.0053 and 0.0512, respectively; Table 1), than for related strains.

**Figure 3:**
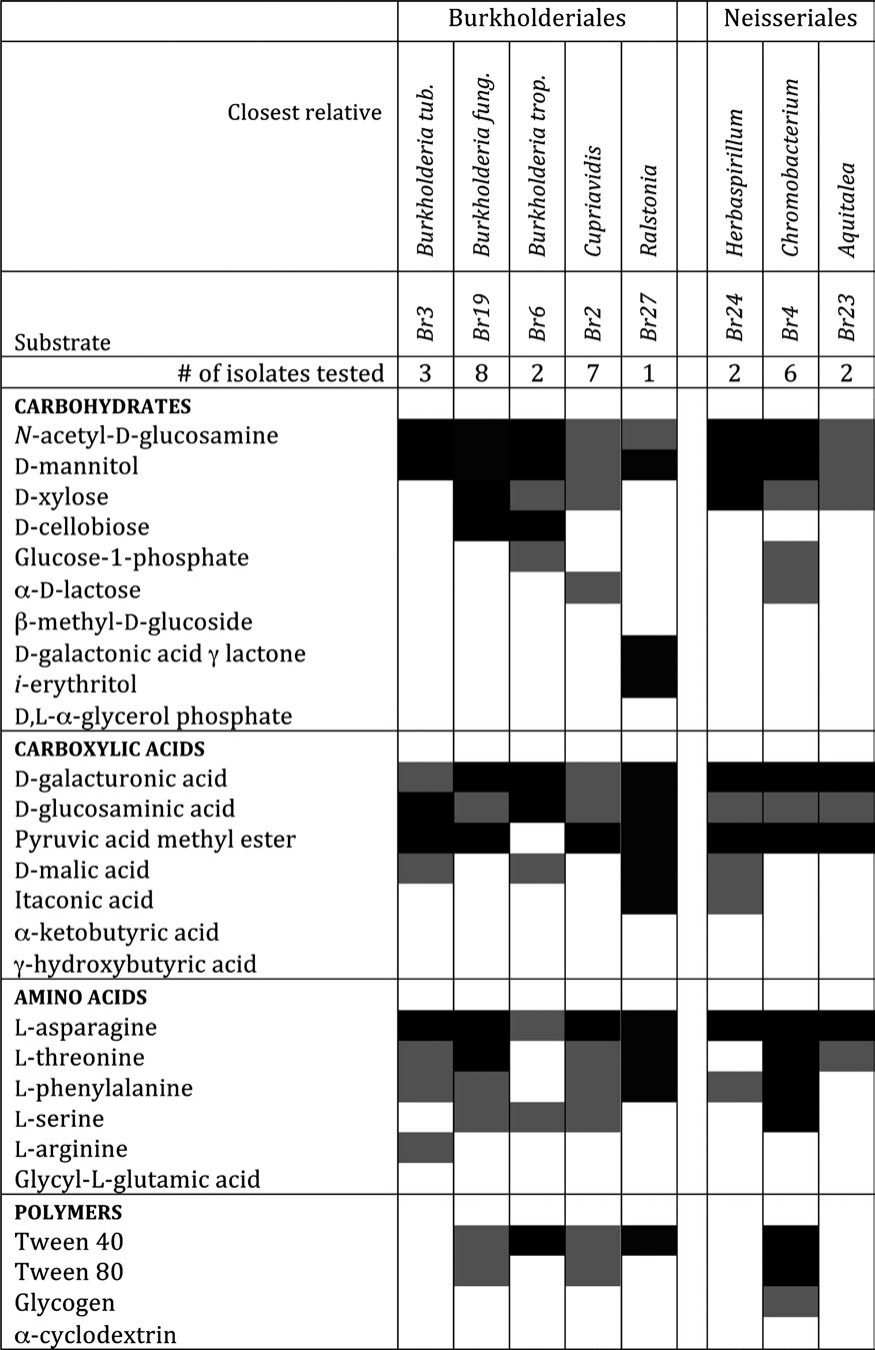
Metabolic profiling for bromeliad‐associated bacterial isolates using 31 defined substrates in six categories (carbohydrates, carboxylic acids, amino acids, polymers, amines, or phenols) with Biolog EcoPlates. Levels of metabolic utilization (as tetrazolium chloride reduction measured spectrophotometrically at 590 nm) were determined for 1–8 isolates of each type, as compared with a sterile water‐only substrate control. Use of amines or phenols was negative for all isolates and, thus, not shown. Black boxes indicate positive values and gray boxes indicate weakly positive values, or capabilities that were variable within isolate types. Empty boxes indicate no detectable activity.

**Figure 4:**
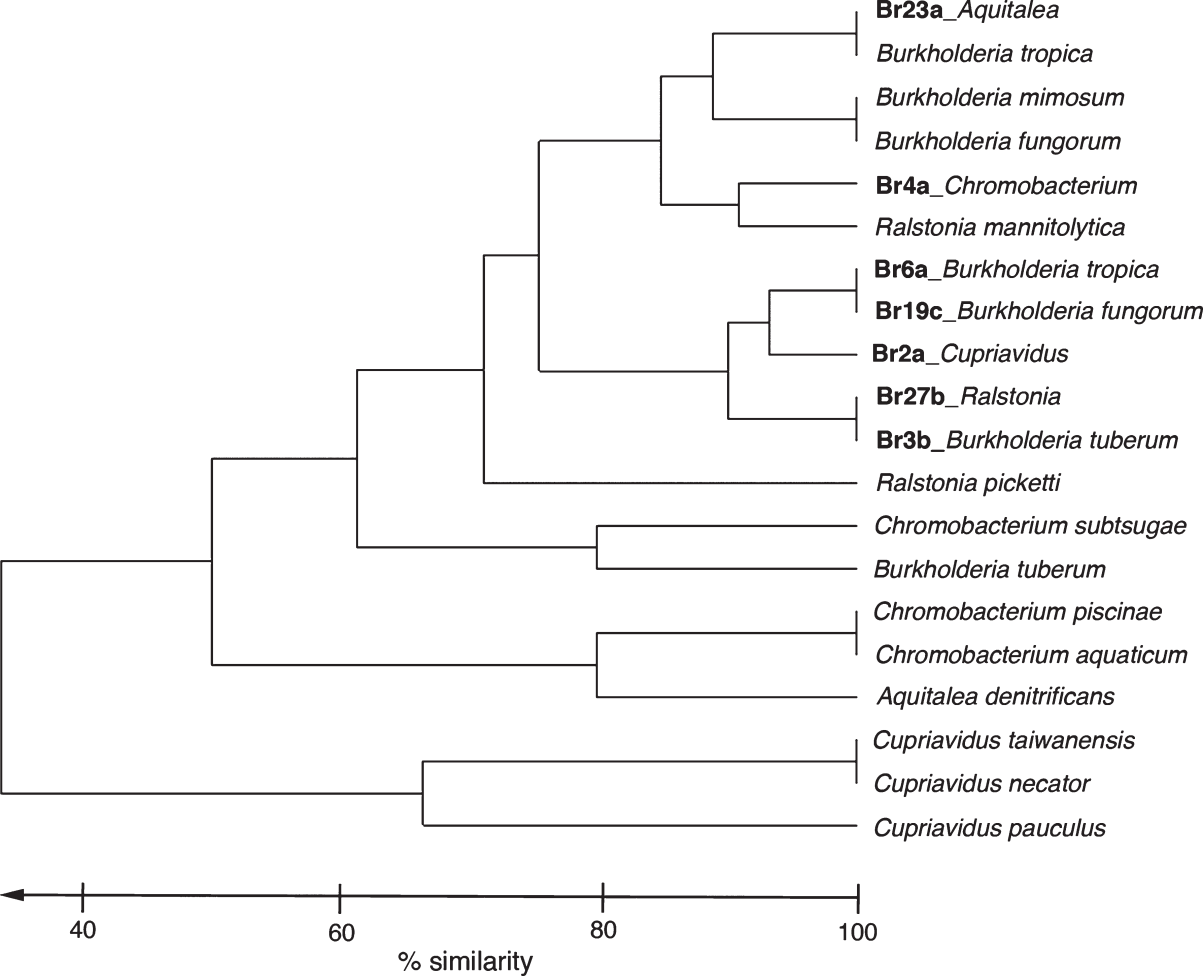
Hierarchical cluster analysis of metabolic phenotypes for bromeliad‐associated isolates recovered in this study, versus strains detailed in other publications. Phenotypic character traits included 19 carbon substrate utilization capabilities, revealed by Biolog EcoPlates, shown in Fig. 3, The analysis, based on Bray–Curtis similarity resemblance (presence/absence, single linkage), was performed using Primer v6.

Isolates of each betaproteobacteria type (*n* = 1–8) were surveyed for their metabolic capabilities (Fig. 3; Tables S1–3). Metabolic activity profiles were overlapping between the Burkholderiales, isolated at pH 4, and the Neisseriales, isolated at pH 6 (Fig. 3). EcoPlate assays revealed the positive use of the following carbon sources by all eight isolate types: d‐galacturonic acid, l‐asparagine, d‐mannitol, *N*‐acetyl‐d‐glucosamine, and d‐glucosaminic Acid (Fig. 3; Tables S1–3). At least six isolate types demonstrated use of pyruvic acid methyl ester, d‐xylose, l‐threonine, and l‐phenylalanine. Fewer than five isolate types utilized Tween 40, Tween 80, l‐serine, and d‐malic acid (Fig. 3; Tables S1–3). The use of mannitol and l‐phenylalanine were both significantly distinct features of the bromeliad‐associated isolates (*P* = 0.0049 and 0.0414, respectively; Table 1), compared to related strains. Hierarchical cluster analysis revealed that bromeliad‐associated isolates were generally distinct from their closest relatives, with regard to carbon utilization profiles (the collective ability to use any of 19 carbon substrates shown in Fig. 3 and 4).

## Discussion

Diverse microorganisms have been recovered from tropical plant sources, including floral nectar, tree holes, *Heliconia* bracts, and bromeliad tank water (Walker et al. 1991; Goffredi et al. 2011a,b; Alvarez‐Pérez et al. 2012; Barbosa et al. 2012). While culture‐based methods are greatly limited in their assessment of the entire microbial community, these findings indicate the presence of a complex aquatic plant microbiota in the rainforest. Tank‐forming, epiphytic plants within the family Bromeliaceae are native to neotropical forests. They have unique foliar tanks that act as basins for rainwater storage, and are home to an array of microbes (Benzing 1970; Bermudes and Benzing 1991; Brighigna et al. 1992; Inselsbacher et al. 2007; Winkler and Zotz 2009; Goffredi et al. 2011a,b). The environment within a bromeliad tank can be acidic (as low as pH 3.5), with suboxic conditions (<0.5 ppm O_2_), and temperatures far above ambient (up to 44°C; Bernal Matarrita, pers comm). Bacterial residents must, therefore, adapt to these conditions in order to survive in this unusual environment. Indeed, most of the betaproteobacteria isolated in this study demonstrated tolerances for low pH and high temperature, as compared with close relatives (Tables 1, S1–S3), and were related to those known to tolerate, or even prefer, low‐oxygen conditions. Betaproteobacteria isolates belonged to heterotrophic groups that either are known to associate with plants or are found in tropical soils or freshwater habitats, including members of the orders Burkholderiales and Neisseriales. In a previous, molecular‐based survey of bacterial communities within Costa Rican bromeliad tanks, Burkholderiales and Neisseriales comprised ~2–7% of the total bacterial community recovered, based on 16S rRNA genes (Goffredi et al. 2011a).

### Bromeliad‐associated Burkholderiales

The presence of the Burkholderiales has been previously observed for bromeliad tanks (Goffredi et al. 2011a). In this study, members of the genus *Burkholderia* (family Burkholderiaceae), in particular, were similarly recovered. Their common association with plants is illustrated by early descriptions of *Burkholderia* as pathogens of onions (at the time named *Phytomonas* spp; Burkholder 1950), yet since then, they have also been recovered from numerous environments, including acidic swamps and tropical soils (Vandamme et al. 2002; Valverde et al. 2003; Yang et al. 2006; Aizawa et al. 2010). The three *Burkholderia*‐related bacterial types isolated from the Costa Rican bromeliad tank cluster within the nonpathogenic “plant‐beneficial‐environmental” clade, and were most closely related to *B. tropica, B. fungorum,* and *B. tuberum* (Fig. 2; Coenye et al. 2001; Ussery et al. 2009; Suárez‐Moreno et al. 2012). These species are generally reported as beneficial for plants both casually and intimately (Reis et al. 2004; Barrett and Parker 2006; Kim et al. 2006; Omarjee et al. 2008; Suárez‐Moreno et al. 2012). *Burkholderia* are known for their versatile ability to degrade recalcitrant carbohydrates and aromatic compounds, such as l‐phenylalanine, and could thus contribute significantly to the turnover of plant‐derived organic carbon that collects in bromeliad tanks (Suárez‐Moreno et al. 2012). Carbon substrate utilization was versatile among the new, and described, *Burkholderia* species, suggesting effective utilization of numerous recalcitrant carbohydrates, including mannitol, cellobiose, xylose, and *N*‐acetyl‐d‐glucosamine (Tables 1, S1).

The sister genera *Ralstonia* and *Cupriavidus* (family Burkholderiaceae) were also recovered in bromeliad tank isolations. Both genera, subdivided nearly 10 years ago based on phenotypic differences, are inhabitants of soil, sludge, and wastewater or associated with plants (Coenye et al. 2003; Vandamme and Coenye 2004; Perez et al. 2008). For example, *Cupriavidus taiwanensis*, closely related to bromeliad isolate *Br2*, is known to be a dominant symbiont of the tropical legume *Mimosa* for which they provide fixed nitrogen, thereby increasing plant growth (Barrett and Parker 2006). *Ralstonia* species closely related to bromeliad isolate *Br27* (ex. *R. pickettii*) have gained significant attention for their ability to degrade recalcitrant compounds (Ryan et al. 2007), and it is possible that similar isolates could contribute significantly to carbon turnover in bromeliad tanks. The *Ralstonia*‐related isolate from bromeliad tank water (*Br27*) was the only member of the genus capable of utilizing mannitol, while the *Cupriavidus*‐related isolate (*Br2*) was the only member of its genus capable of utilizing mannitol, xylose, and *N*‐acetyl‐d‐glucosamine, consistent with the exploitation of a habitat rich in plant‐based materials (Tables 1, S2).

Similarly, members of the genus *Herbaspirillum* (order Burkholderiales, family Oxalobacteraceae), which were recovered in two tank water isolation attempts, are primarily associated with the root surfaces and tissues of plants, for which they have been shown to promote growth (Baldani et al. 1986; Kirchhof et al. 2001; Valverde et al. 2003). As a genus, they are generally capable of utilizing plant‐sourced carbon substrates such as d‐galacturonic acid, mannitol, xylose, and *N*‐acetyl‐d‐glucosamine (Rothballer et al. 2006), as was the close relative isolated in this study, *Br24* (Fig. 3).

### Bromeliad‐associated Neisseriales

Bacteria within the genera *Chromobacterium* and *Aquitalea* (family Neisseriaceae) were isolated in this study at pH 6. Members of the genus are found in tropical and subtropical freshwater and terrestrial environments (Brazilian National Genome Project Consortium 2003; Martin et al. 2007; Young et al. 2008; Kampfer et al. 2009). The closest relative of the bromeliad‐associated isolate *Br4*,* Chromobacterium subtsugae*, has been recovered from soil rich in hemlock leaves and metabolizes plant compounds such as galacturonic acid and l‐phenylalanine (Martin et al. 2007). Species within this relatively new genus vary in their ability to live in anoxic environments (Leifson 1956). Well‐developed bromeliad tanks are highly stratified, with dramatically higher oxygen within 1 cm of the surface of the tank (up to 8 ppm O_2_), compared to <1 ppm O_2_ in the bottom (Goffredi et al. 2011b). It is, therefore, possible that *Chromobacterium*‐related members of the tank community inhabit the upper layers of the basin where not only higher O_2_ conditions persist, but also higher pH conditions (by as much as 1 pH unit; Goffredi et al. 2011b). Exclusively, bromeliad isolates related to *Chromobacterium* were recovered in this study at pH 6 and were unable to proliferate at pH 4. Nevertheless, isolate *Br4* had a pH range shifted slightly lower, as well as the unique capability to metabolize d‐mannitol and xylose, compared to other members of *Chromobacterium* (Tables 1, S3). Members of the genus *Aquitalea* (family Neisseriaceae) are facultative anaerobes isolated previously from similarly organic‐rich environments, such as humic lakes and wetland peat (Lau et al. 2006; Lee et al. 2009). They are distinct from *Chromobacterium* in their inability to use l‐phenylalanine and their reduced tolerance to higher salinity and temperatures. The *Aquitalea‐*related isolate *Br23* from this study uniquely displayed the ability to metabolize starch, d‐galacturonic acid, mannitol, xylose, and *n*‐acetyl‐d‐glucosamine (Tables 1, S3).

### Community function: breakdown of organic substrates

Bromeliads, with their unique ∨‐shaped central rosette and trough‐like leaves, are known to collect large amounts of both plant‐ and animal‐derived material (Benzing et al. 1972; Pittl et al. 2010). As a consequence, these catchments are an ideal site for microbial decomposition. The ability of all bacterial isolates examined in this study to utilize plant‐derived compounds indicates their likely facilitation in the turnover of leaves and plant debris entrained within the tanks from the overlying rainforest canopy. For example, all isolate types could use d‐galacturonic acid, a component of the plant cell wall (Isherwood et al. 1953) and mannitol, a sugar alcohol produced by plants during times of osmotic stress to balance inner solute concentrations (Stoop et al. 1996). The ability to use mannitol was confirmed using APIZYM analysis and growth in phenol red fermentation agar (data not shown). Similarly, six isolate types were capable of using d‐xylose, a key component of pectin (Mohnen 2008) and three isolate types used l‐phenylalanine, which is synthesized by chloroplasts, and serves as a precursor to many plant‐derived metabolites (Jung, 1986). The ability to use these substrates was unique, in some cases, compared to close relatives (Table 1), and may prove to be an important component for the niche specialization of bacteria within the bromeliad tank.

A further capability demonstrated by all eight isolates was the use of *N*‐acetyl‐d‐glucosamine, a monomeric unit of chitin. While not a unique trait, these bacteria do appear to be poised to also aid in the breakdown of chitinous remains of insect exoskeletons, and fungal cell walls, prevalent within the bromeliad tank environment. Chitinases have been previously recovered from bromeliad tanks, including one from *Chromobacterium* (Goffredi et al. 2011a), confirming the likely role of microbial residents in the biological remineralization of recalcitrant chitin in tropical forests. The ability to use *N*‐acetyl‐d‐glucosamine and/or chitin directly was confirmed using APIZYM analysis and growth in phenol red fermentation agar (data not shown).

It is important to note that fungi are also likely to play an important role in the hydrolysis of plant material trapped in bromeliad tanks. In a previous metatranscriptomic analysis, genes from eight fungal classes, including Ascomycota and Basidiomycota, were recovered from the water catchments formed by Costa Rican bromeliads (Goffredi et al. 2015). It will therefore be important, in the future, to examine the likely involvement of fungi in the stability and functioning of bromeliad ecosystems.

## Conclusion

Betaproteobacteria from two orders were specifically isolated from the unique aquatic habitat within a bromeliad tank. Compared to close relatives, the isolates displayed high temperature and comparatively low pH optima, reflecting the tropical, acidic nature of bromeliad tanks. Metabolic activity profiles were aligned with a role in the breakdown of plant‐sourced material. Further, these activities overlapped between the Burkholderiales, isolated at pH 4, and the Neisseriales, isolated at pH 6, suggesting that plant material decomposition occurs in tanks of both pH extremes. Despite their importance within neotropical habitats, the specific nutritional strategy used by bromeliads remains a mystery. We suggest that future efforts focus on expanding current knowledge on the specific role of bacterial, as well as fungal, mineral leeching, nitrogen fixation, and metabolic exchanges with these important ecosystem‐structuring plants.

## Conflict of Interest

The authors declare no conflict of interest in relation to the submitted work.

## Supporting information


**Table S1.** Phenotypic characteristics of bromeliad tank strains in comparison to other closely related members of the genus *Burkholderia*.
**Table S2.** Phenotypic characteristics of bromeliad tank strains in comparison to other members of the genera *Ralstonia* and *Cupriavidus*.
**Table S3.** Phenotypic characteristics of bromeliad tank strains in comparison to other members of the genera *Chromobacterium* and *Aquitalea*.
