# Supplementary material for: Decomposition of plant‐sourced carbon compounds by heterotrophic betaproteobacteria isolated from a tropical Costa Rican bromeliad
Source: Microbiologyopen. 2016 Feb 25;5(3):479–89. doi: 10.1002/mbo3.344 (PMC4905999; doi:10.1002/mbo3.344)
Supplement: Supplementary file 1 — Table S1. Phenotypic characteristics of bromeliad tank strains in comparison to other closely related members of the genus Burkholderia. Table S2. Phenotypic characteristics of bromeliad tank strains in comparison to other members of the genera Ralstonia and Cupriavidus. Table S3. Phenotypic characteristics of bromeliad tank strains in comparison to other members of the genera Chromobacterium and Aquitalea. [file MBO3-5-479-s001.pdf]

Table 1 | Phenotypic characteristics of bromeliad tank strains in comparison to other closely related members of the genus *Burkholderia*

Characteristic	***Br3***	*B. tuberum*	***Br19***	*B. fungorum*	***Br6***	*B. tropica*	*B. mimosarum*
Isolation source	bromeliad tank	nodules of legumes	bromeliad tank	white rot fungus	bromeliad tank	rhizosphere	nodules of legumes
Temp (°C)	23-42	28-30	23-42	25-37	23-42	22-37	28-37
pH Range	4-7.5	na	4-7	na	4-7	5-7	na
Size (µm)	1-1.5	na	2-3	na	1.5-2.0	0.7-1.5	0.8-2.0
Oxidation of:							
Starch	nm	+	+	na	-	-	na
Casein	nm	na	+	na	+	na	na
D-Mannitol	+	+	+	+	+	+	+
D-Galacturonic Acid	(+)	na	+	na	+	+	+
D-Xylose	-	na	+	(+)	(+)	(+)	+
D-Cellobiose	-	na	+	na	+	(+)	-
L-Phenylalanine	(+)	na	(+)	na	(+)	+	+
N-Acetyl-D-Glucosamine	+	+	+	+	+	(+)	+

Strain references: *B. tuberum* (Vandamme et al. 2002). *B. tropica* (Reis et al. 2004; Aizawa et al. 2010), *B. mimosarum* (Chen et al. 2006), *B. fungorum* (Coenye et al. 2001), *B. phytofirmans* (Sessitsch et al. 2005). `+´, none of the isolates tested gave a positive reaction. `-´, none of the isolates tested gave a positive reaction. `(+)´ = isolates showed variable reactivity, or reaction was weak. `na´ = not available from previous studies. `nm´ = not measured in this study.

Table 2 | Phenotypic characteristics of bromeliad tank strains in comparison to other members of the genera *Ralstonia* and *Cupriavidus*

Characteristic	***Br2***	*C. necator*	*C. pauculus*	*C. taiwanensis*	***Br27***	*R. picketti*	*R. mannitolytica*
Isolation source	Bromeliad tank	Soil	Groundwater	Root nodules of *Mimosa*	Bromeliad tank	Clinical sources	Clinical sources
Temp (°C)	23-47	27-37	30-42	28-37	23-42	30-42	30-42
pH Range	4-7	5.5-9.2	na	na	4-7.5	na	na
Size (μm)	2-5	0.7-1.3	1-2	0.5-2.0	1-2	na	na
Oxidation of:							
Starch	+	-	-	na	-	na	na
Casein	+	na	na	na	nm	na	(+)
D-Mannitol	(+)	-	-	-	+	-	+
D-Galacturonic Acid	(+)	na	na	na	+	na	na
D-Xylose	(+)	-	-	-	-	(+)	+
D-Cellobiose	-	na	na	na	-	na	na
L-Phenylalanine	(+)	na	na	(+)	+	+	(+)
N-Acetyl-D-Glucosamine	(+)	na	-	-	(+)	+	+

Strain references: *C. necator* (Makkar and Casida 1987); *C. pauculus* (Vandamme et al. 1999; Vaneechoutte et al. 2004); *C. taiwanensis* (Chen et al. 2001; Vaneechoutte et al. 2004); *R. picketti* (Coenye et al. 2003); *R. mannitolytica* (De Baere et al. 2001; Coenye et al. 2003). `+´, none of the isolates tested gave a positive reaction. `-´, none of the isolates tested gave a positive reaction. `(+)´ = isolates showed variable reactivity, or reaction was weak. `na´ = not available from previous studies.

Table 3 | Phenotypic characteristics of bromeliad tank strains in comparison to other members of the genera *Chromobacterium* and *Aquitalea*

Characteristic	***Br4***	*C. subtsugae*	*C. aquaticum*	*C. violaceum*	*C. piscinae*	***Br23***	*A. denitrificans*
Isolation source	Bromeliad tank	Temperate soil	Spring water	Soil Water	Pond water	Bromeliad tank	Wetland soil
Temp (°C)	23-42	10-37	32[a]	25-28	32[a]	23-37	10-40
pH Range	5.0-7.5	6.5-8.0	5-9.5	6.5-8.0	5-9.5	5.0-7.5	5-9
Size (μm)	2-3	2.2-2.6	1.5-2	na	0.3-2	2-3	0.7-2.5
Oxidation of:							
Starch	(+)	na	na	na	na	+	-
Casein	+	+	na	+	na	-	-
D-Mannitol	+	-	-	-	-	(+)	-
D-Galacturonic Acid	+	na	+	na	na	+	na
D-Xylose	(+)	-	-	na	-	(+)	na
D-Cellobiose	-	-	(+)	na	-	-	na
L-Phenylalanine	+	-	(+)	(+)	-	-	na
N-Acetyl-D-Glucosamine	+	+	+	na	+	(+)	+

Strain references: *C. subtsugae* (Martin et al. 2007); *C. aquaticum* (Young et al. 2008; Kampfer et al. 2009); *C. violaceum* (Martin et al. 2007; Young et al. 2008); *C. piscinae* (Kampfer et al. 2009), *A. denitrificans* (Lee et al. 2009). `+´, none of the isolates tested gave a positive reaction. `-´, none of the isolates tested gave a positive reaction. `(+)´ = isolates showed variable reactivity, or reaction was weak. `na´ = not available from previous studies. [a]No range given.
